# Morroniside maintains mitochondrial homeostasis in microglia and mitigates neuroinflammation in experimental autoimmune encephalomyelitis

**DOI:** 10.1093/jnen/nlag038

**Published:** 2026-04-20

**Authors:** Shaopeng Zhai, Taotao Jiang, Xiaorong Cheng, Xin Li, Qi Wang, Qing Fu, Boyao Yuan, Fengjuan Wu, Manxia Wang

**Affiliations:** Department of Neurology, Lanzhou University Second Hospital, Lanzhou, China; Department of Neurology, Lanzhou University Second Hospital, Lanzhou, China; Department of Rehabilitation, Lanzhou University Second Hospital, Lanzhou, China; Department of Neurology, Lanzhou University Second Hospital, Lanzhou, China; Department of Neurology, Lanzhou University Second Hospital, Lanzhou, China; Department of Rehabilitation, The First Hospital of Lanzhou University, Lanzhou, China; Department of Neurology, Lanzhou University Second Hospital, Lanzhou, China; Department of Neurology, Lanzhou University Second Hospital, Lanzhou, China; Department of Neurology, Lanzhou University Second Hospital, Lanzhou, China

**Keywords:** experimental autoimmune encephalitis (EAE), microglia, mitochondrial homeostasis, mitophagy, morroniside, Nrf2

## Abstract

Microglia-mediated neuroinflammation in the central nervous system is a hallmark of both multiple sclerosis (MS) and the animal model experimental autoimmune encephalomyelitis (EAE). Current immunosuppressive therapies for MS have limited efficacy and notable side effects. This study aimed to investigate mechanisms underlying anti-neuroinflammatory effects of morroniside, an iridoid glycoside derived from *Cornus officinalis*, which is used in Chinese herbal medicine. Morroniside treatment significantly attenuated lipopolysaccharide-induced alterations and mitochondrial dysfunction in BV2 microglia cells. *In vivo*, morroniside treatment improved clinical scores and ameliorated pathological findings and neurological deficits in a mouse EAE model. Mechanistic investigations revealed that morroniside activated the Nrf2/HO-1 signaling axis, promoted Nrf2 nuclear translocation and elevated *HO-1* expression. This activation also upregulated *p62,* thereby enhancing LC3-II/PINK1/Parkin-mediated mitophagosome formation. The resultant mitophagy suppressed p65 phosphorylation leading to anti-inflammatory effects. Collectively, our findings suggest that morroniside ameliorates EAE by increasing anti-inflammatory microglial activation through upregulating the Nrf2/HO-1/p62 axis to mitigate mitochondrial oxidative stress and enhance mitophagy. These results identify morroniside as a promising therapeutic candidate for MS and emphasize the importance of the Nrf2-p62-mitophagy axis in resolving neuroinflammation and maintaining mitochondrial homeostasis.

## Introduction

Multiple sclerosis (MS) is an autoimmune inflammatory neurodegenerative disease, characterized by immune cell infiltration into the central nervous system (CNS), demyelination, axon degeneration, and neuronal loss.^1^ The clinical symptoms of MS include visual impairment, limb weakness, paresthesia, ataxia, autonomic dysfunction, and cognitive dysfunction. With a rising global prevalence^2^ and high personal and socioeconomic burden, MS poses a growing global health challenge. Current disease-modifying therapies such as fingolimod have shown limited efficacy in progressive MS patients with some of these therapies producing notable side effects.^3^ Although the exact molecular mechanism of MS remains unclear, several pathological processes, such as abnormal microglial activation, excessive oxidative stress, and mitochondrial dysfunction, are recognized as important contributors to the pathogenesis of MS and its animal model, experimental autoimmune encephalomyelitis (EAE). The EAE animal model clearly recapitulates MS and is widely used to study MS.^4^

Microglia are innate immune cells resident in the CNS that have a pivotal yet dual role in the pathogenesis of MS and EAE. In the initial stage of disease, microglia have a pro-inflammatory phenotype induced by interferon-gamma (IFN-γ), lipopolysaccharide (LPS), and tumor necrosis factor-alpha (TNF-α); they release pro-inflammatory cytokines that promote inflammatory responses and demyelination. Conversely, in the later disease stage, microglia act in an anti-inflammatory manner.^5^^,^^6^ Targeting microglia to modulate their dual inflammatory roles is a vital strategy for alleviating neuroinflammation notwithstanding the involvement of other cell types in the MS and EAE.

Oxidative stress, fueled by reactive oxygen and nitrogen species (ROS and RNS) that are released from damaged mitochondria in pro-inflammatory microglia, is a well-established driver of MS and EAE pathology.^7^^,^^8^ Oxidative stress not only contributes to demyelination, neurodegeneration, and mitochondrial dysfunction by oxidizing proteins, DNA, and lipids but also acts as a signaling molecule that activate pro-inflammatory pathways, thereby initiating a self-perpetuating “oxidation-inflammation” cycle.^9–11^ The nuclear factor erythroid 2-related factor 2 (Nrf2), an important mediator of antioxidant signaling,^12^ may disrupt this “oxidation-inflammation” cycle. Emerging evidence suggests that the core therapeutic benefits of Nrf2 activation may lie in its ability to alleviate mitochondrial damage.^13^

Morroniside, a prominent iridoid glycoside compound derived from the traditional Chinese herbal medicine *Cornus officinalis*, has demonstrated neuroprotective effects in several oxidative stress-related neurological disorder models, including Alzheimer disease,^14^ Parkinson disease,^15^ and ischemic stroke,^16^^,^^17^ primarily by activating antioxidant pathways. However, its potential effects in EAE, particularly whether it modulates microglial-driven neuroinflammation, remain unexplored. We hypothesized that morroniside alleviates EAE by upregulating Nrf2 signaling, which enhances mitophagy and attenuates oxidative stress, thereby attenuating pro-inflammatory microglia activation and ultimately ameliorating neuroinflammation.

## Methods

### Animals

Female C57 BL/6J mice were purchased from the Medical Experimental center of Lanzhou University and ranged from 8 to 10 weeks with a mean weight 20 ± 2 g. Mice were housed in the Cuiying Biomedical Research Center, Lanzhou University Second Hospital, which was a specific pathogen-free (SPF) environment. We provided enough food, purified water and consistent 12-hour light–dark cycle to mice.

### EAE induction and morroniside treatment

For EAE induction, mice (10 to 12 weeks old) were immunized subcutaneously with 300 μg MOG35–55 peptide (GL Biochem, China) emulsified in complete Freund’s adjuvant (CFA) supplemented with 10 mg/mL *Mycobacterium tuberculosis* H37RA (Sigma-Aldrich, USA). The mice received intraperitoneal injections with 250 ng pertussis toxin (List Biological Labs, USA) at the time of immunization and 48 hours later.^18^ Morroniside (CAS NO. 25406-64-8, ChemFaces) was administrated intragastric once per day from day 7 to day 21 post-immunization (p.i.). The control and EAE group were injected intraperitoneally with vehicle in the same manner.

### Behavioral tests

#### Neurological symptom scores and weight changes

Two trained researchers independently recorded body weight changes and neurological symptom scores under double-blind conditions for 21 consecutive days. The neurological scoring criteria were defined as follows: 0—normal, asymptomatic; 1—tail weakness; 2—inability to right itself; 3—complete hindlimb paralysis; 4—tetraplegia; 5—moribund state or death.

#### Open-field test (OFT)

Open-field test (OFT) was used to evaluate spontaneous activity of mice.^19^ The mice were placed in the center of the open field for 5 minutes and the total distance was recorded. After each experiment, we cleaned the open field with 75% ethanol solution to ensure the next mouse would not be affected by any odor left.

#### Beam walking

The beam walking test was performed to evaluate the motor function of mice.^20^ The beam walking test apparatus consisted of a long strip of wood (length of 100 cm, width of 0.6 cm). The beam was suspended at a 60 cm height. The mice were placed at the initial end of the beam and were trained to cross the beam in 60 seconds. The time used to across the beams and number of foot slips were recorded for each test.

### BV2 microglia culture

BV2 mouse microglia (Pricella, China) were cultured in high-glucose DMEM (BasalMedia, China) with 10% fetal bovine serum (FBS) (BS-1105, OPCEL, China). Cells were maintained in a cell incubator (Heal Force, China) at 37°C with 5% CO_2_ and 95% air.

### Cell counting kit-8 assay (CCK-8)

Cell counting kit-8 assay (CCK-8) assay (Solarbio, China) was used to access BV2 microglia viability incubated with LPS (1, 3, 5, 9, 15 μg/mL) or morroniside (1, 5, 10, 50, 100, 150 μM). After 24 hours of treatment, 10 μL of CCK-8 regent was added to cells and incubated at 37°C for 30 minutes. Absorbance was read at 450 nm using microplate reader (Biotek, USA).

### Network pharmacology analysis of morroniside

To explore the potential targets and mechanisms of morroniside in anti-neuroinflammation, a network pharmacology approach was employed. First, the canonical SMILES structure of morroniside (retrieved from PubChem: CID 11228693) was submitted to Swiss Target Prediction, SuperPred,s and PharmMapper databases for target prediction. Overlapping targets from both platforms were selected to ensure reliability. Disease-associated genes related to “multiple sclerosis” was collected from Genecards, DisGeNET, OMIM and Therapeutic Target Database databases. The intersection between morroniside-predicted targets and disease-related genes was visualized using a Venn diagram. Protein-protein interaction (PPI) networks were constructed via the STRING database (v11.5) and analyzed in Cytoscape (v3.9.1) to identify hub targets based on degree centrality. Functional enrichment analysis for Gene Ontology (GO) terms and Kyoto Encyclopedia of Genes and Genomes (KEGG) pathways was performed using DAVID (v6.8) with false discovery rate (FDR) correction (*P < .*05).

### Immunofluorescence analyses

BV2 cells after treatment were washed with PBS, fixed with 4% paraformaldehyde (Servicebio, China) for 15 minutes and then permeabilized with 0.1% Triton X-100 (Solarbio) for 10 minutes. For tissue immunofluorescent, mice were killed on the day 21 post-immunization and immediately perfused with saline followed by 4% paraformaldehyde, then the brain and lumbar cord tissues were subsequently harvested. Tissues samples were fixed in 4% paraformaldehyde for 24 hours, dehydrated through a graded ethanol series (70%, 80%, 95%, and 100%), and embedded in paraffin. Sections of 5 μm thickness were cut using a microtome (Leica, Germany) and mounted on glass slides. Cells or tissue slices were blocked with 5% skim milk for 1 hour, then incubated overnight at 4°C with primary anti-Nrf2 antibody (Affinity; AF0639; 1:250), primary anti-LC3 antibody (Affinity; AF4650; 1:250), primary anti-TOM20 antibody (Proteintech; 66777-1-Ig; 1:250), primary anti-CD86 antibody (Affinity; DF6332; 1:250), primary anti-CD206 antibody (Affinity; DF4149; 1:300), primary anti-TFAM antibody (HUABIO; ET1705-64; 1:250), primary anti-Iba1 antibody (Oasis; OB-PGP049-0; 1:500), and primary anti-MBP antibody (Affinity; AF4085; 1:250). The next day, the slices were washed by PBS and incubated with secondary antibodies goat anti-rabbit IgG (Affinity; S006; 1:200) and goat anti-mouse IgG (Affinity; S0017-100; 1:200) for 1 hour in dark. Nuclei were stained with DAPI (Biosharp, China). Images were captured using inverted fluorescent microscope (OLYMPUS, Japan) and TissueFAXS Cytometry-Based Quantitative Analysis System (Tissue Gnostics Asia Pacific Limited, Austria).

### Nrf2 siRNA transfections

Small interfering RNA (GenePharma, China) was used to knockout Nrf2. Before transfection, BV2 cells were cultured at 37°C in the incubator until 50% to 60% confluent. Next, 5 μL of Nrf2 siRNA duplex was diluted into 200 μL of DMEM. The dilutions were mixed gently and incubated for 5 minutes. Then 3 μL of GP-transfect-Mate (GenePharma) was diluted into the dilutions and mixed gently for 10 minutes. Next, cells were incubated in Nrf2 siRNA transfection cocktail with DMEM for 24 to 48 hours at 37°C. Transfection efficiency was accessed by quantitative real-time PCR (qRT-PCR) and Western blot (WB) for levels of Nrf2 gene and protein expression.

### Quantitative real-time PCR (qRT-PCR)

Total RNA was extracted from BV2 cells, brains, and spinal cords using Trizol reagent (AG21101, Accurate Biology). The cDNA synthesis was performed using Evo M-MLV RT mix for qPCR (AG11705, Accurate Biology). qRT-PCR was performed using Pro Taq HS Premix Probe qPCR kit (AG11704, Accurate Biology) on the CFX96 system (Biorad, USA). The relative gene expression ratios were calculated by using the 2^−ΔΔCt^ method. The primer sequences are listed in Table 1.

**Table 1:** Primer sequences used for qPCR.

Gene name	Forward primers	Reverse primers
*II6*	TGCCTTCTTGGGACTGAT	CTGGCTTTGTCTTTCTTGTT
*Tnf*	CCACCACGCTCTTCTGTCTACTGA	GACGGCAGAGAGGAGGTTGACTT
*II10*	CACTGCTATGCTGCCTGCTCTT	CTTCACCTGCTCCACTGCCTTG
*II1b*	GTGTCTTTCCCGTGGACCTT	AATGGGAACGTCACACACCA
*Nfe2l2*	GCACGCTGAAGGCACAATGGA	GGATTCACGCATAGGAGCACTGT
*Hmox1*	GACCGCCTTCCTGCTCAACATT	CTCTGACGAAGTGACGCCATCTG
*Gapdh*	TGTGTCCGTCGTGGATCTGA	TTGCTGTTGAAGTCGCAGGAG

All primers were designed based on the reference sequences from NCBI GenBank.

### Western blot (WB)

Cells, brain, and spinal cord were lysed by RIPA lysate (Solarbio, China). The protein extracted were separated by SDS-PAGE and transferred to PVDF membranes (10600023, Cytiva, USA). Membranes were blocked with 10% skim milk for 1 hour at room temperature and then incubated with primary antibodies at 4°C overnight: anti-Nrf2 antibody (Affinity; AF0639; 1:5000), anti-HO-1 antibody (Proteintech; 66743-1-Ig; 1:3000), primary anti-LC3 antibody (Affinity; AF4650; 1:3000), primary anti-p62 antibody (Affinity; AF5384; 1:3000), primary anti-PARKIN antibody (Proteintech; 66674-1-Ig; 1:2000), primary anti-PINK1 antibody (Affinity; DF7742; 1:2000), primary anti-phosphorylated p65 (p-65) antibody (Wanleibio; WL02169; 1:1000), primary anti-p-IκB antibody (Wanleibio; WL02495; 1:1000). Next day, the membranes were washed using Tween 20 Tris buffer, and incubated with secondary antibodies for 1 hour. The protein expression levels were visualized using an Automatic chemiluminescence image processing system (Tanon 5200 Multi). The intensity of protein bands was quantified by Fiji 2.

### Enzyme-linked immunosorbent assay

The cytokine protein levels were accessed using enzyme-linked immunosorbent assay (ELISA) kit. We collected brain tissues and determined the concentrations of TNF-α (AG-20B-0081PF-C100, NEOBIOSCIENCE), IL-6 (BE0046-1MG, NEOBIOSCIENCE), and IL-10 following the manufacturer’s instructions. Absorbance was measured at 450 nm by microplate reader.

### Measurement of SOD and MDA levels

Intracellular SOD and MDA levels were measured by SOD assay kit (A001-1-1, Nanjing Jiancheng Bioengineering Institute) and MDA assay kit (A003-4-1, Nanjing Jiancheng Bioengineering Institute) separately. The supernatant was collected and the assay was used following the manufacturer’s instructions.

### NO measurement

NO production was accessed by detecting nitrite levels in BV2 cell culture supernatants. using NO assay kit (S0020S, Beyotime) following the manufacturer’s instructions. NO levels were quantified through a nitrite standard solution.

### Mitochondrial DNA (mtDNA) copy number detection

The mtDNA copy number was performed to determine the number of mitochondrial. After treatment, BV2 cells were harvested and extracted total DNA using a Genomic DNA Extraction Kit (Accurate BIOLOGY). The amount of mitochondrial relative to nuclear DNA was accessed by qRT-qRT-PCR using primer for Nd2 and GAPDH (Tsingke Biotechnology Co., Ltd.).

### ROS measurement

Intracellular ROS levels were measured by Reactive Oxygen Species Detection Kit (Beyotime, China). After treatment, BV2 cells were harvested and stained with membrane-permeable fluorescent probe 2’,7’-dichlorofluorescein (DCFH-DA) at a dilution of 1:1000 for 20 minutes. Then, we washed the cells by PBS and harvested cell precipitation after centrifugation. The cells were resuspended with PBS. Microglia surface staining was assessed using flow cytometry (Beckman Coulter, USA). ROS levels were accessed using the mean fluorescence intensity.

### JC-1 measurement

Mitochondrial membrane potential was measured by using JC-1 Detection Kit (Beyotime, China). Cells were harvested and stained with JC-1 fluorescent probe 2’,7’- at a dilution of 1:200 for 20 minutes according to the manufacturer’s instructions. Then, we washed the cells and harvested cell precipitation after centrifugation. The cells were resuspended with PBS. Cell surface staining was assessed by flow cytometry.

### Mitochondrial ROS measurement

Mitochondrial ROS levels were measured by MitoSOX Red kit (Thermo Fisher Scientific, USA) according to the manufacturer’s instructions. Firstly, treated cells cultured on glass bottom dishes were washed by PBS, and then incubated with 0.5 μM MitoSOX probe. Then cells were washed and incubated with Hochest33422 stain solution (C0030, Solarbio) for 5 minutes. Then we washed the cells and used confocal microscopy to detect superoxide anion.

### Transmission electron microscopy (TEM)

BV2 cells were harvested in 1 mm^3^ precipitation and then fixed in 2.5% glutaraldehyde. The samples were refixed by 1% Osmium tetroxide and dehydrated by Gradient acetone. Next, the samples were embedded in Epom-812 medium for 24 hours. Then we used an ultra-thin microtome to make 70 nm ultrathin slices and fish them onto the copper mesh. The slices were stained by uranium acetate for 10 minutes, and then lead citrate for 2 minutes. We observed the mitochondrial morphology and mitophagosomes using TEM (JEM 1011, Japan).

### Flow cytometric analysis of CD86/CD206

After treatment, BV2 microglia were harvested and stained with PE-conjugated anti-CD86 (B7-2, Themo Fisher Scientific) and permeabilization buffer (GAS005, MΜLTI Science) for 20 minutes. Then, we washed the cells by PBS and harvested cell precipitation after centrifugation. The cells were resuspended with permeabilization buffer and stained with APC-conjugated anti-CD206 (17 to 2061-82, Themo Fisher) for 1 hour. We washed and suspended the cells by PBS after centrifugation. Cell surface staining was assessed using flow cytometry.

### H&E staining

The lumbar cord slides were dewaxed in xylene solution and rehydrated through a descending ethanol gradient to distilled water. Nuclei were stained with hematoxylin (G1120, Solarbio, China) for 10 minutes, followed by rinsing in distilled water to remove excess dye. Cytoplasmic counterstaining was performed with eosin (G1120, Solarbio, China) for 2 minutes. Dehydration was accomplished through an ascending ethanol series. Finally, sections were cover slipped and observed under a light microscope. Inflammation was scored as Okuda pathological grading system.^21^

### Statistical analysis

All data were expressed as the mean ± SEM (standard error of the mean). Shapirp-Wilk test was used to test if the individual data were normally distributed. One-way analysis of variance (ANOVA) or 2-way ANOVA followed by Tukey’s *post hoc* tests were used for comparisons among multiple groups. Values of *P < .*05 was considered statistically significant. Graphs were performed using GraphPad Prism 10 (GraphPad Software Inc., USA).

## Results

### Network pharmacology reveals multi-target mechanisms of morroniside

The canonical SMILES structure of morroniside is shown in Figure 1A. A network pharmacology analysis identified 10 995 potential targets of morroniside, 226 of which overlapped with MS-related disease genes (Figure 1B). A PPI network analysis highlighted 23 hub targets, including Nrf2 (*NFE2L2*) and NF-κB (*RELA*), which exhibited high connectivity (degree > 15) (Figure 1C). An enrichment analysis revealed significant associations with the inflammatory response (GO: 0006954, FDR = 5.74 × 10^−7^), Nrf2 signaling pathway (GO: 00045944, FDR = 1.84 × 10^−5^), response to reactive oxygen species (KEGG: 05208, FDR = 2.75 × 10^−6^), and pathways of neurodegeneration—multiple diseases (KEGG: 05022, FDR = 0.011057483) (Figure 1D and E). Additionally, our previous study found that the NF-κB signaling axis was critically involved in EAE pathogenesis and progression.^22^ Therefore, we hypothesized that morroniside alleviates EAE by orchestrating a multi-target mechanism that restores oxidation and suppresses neuroinflammation.

**Figure 1:**
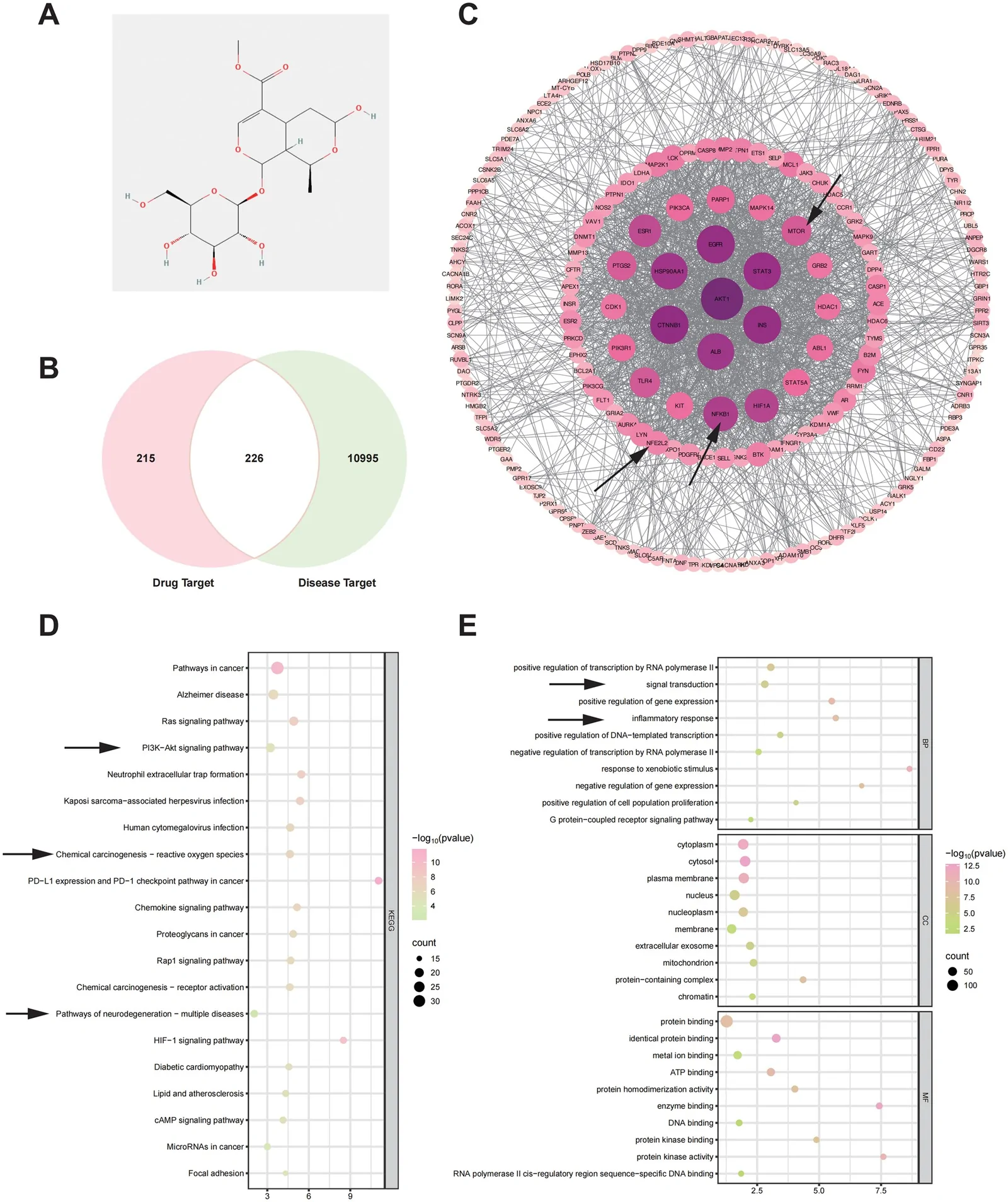
Network pharmacological analysis of MOR against MS. (A) The canonical SMILES structure of morroniside. (B) Venn diagram showing the relevant target genes for MS and MOR. (C) Target-gene interaction PPI network. (D) KEGG analysis of MOR against MS. (E) GO enrichment analysis of MOR on MS-predicted proteins.

### Morroniside alleviates the LPS-induced inflammatory response and oxidative stress in BV2 microglia *in vitro*

To investigate the underlying anti-inflammatory mechanism of morroniside *in vitro*, we used LPS-stimulated BV2 microglia, a well-established cell model for studying neuroinflammation. First, the impact of morroniside and LPS on cell viability was assessed by a CCK-8 assay. We observed that morroniside at concentrations below 50 μM and LPS at concentrations below 3 μg/mL had no significant effect on cell viability (Figure 2A and B). Consequently, BV2 cells were co-treated with 500 ng/mL LPS and various concentrations of morroniside (1, 10, 50, and 100 μM) for 24 hours in subsequent experiments. Morroniside significantly suppressed LPS-induced nitric oxide (NO) production (Figure 2C). At the transcriptional level, morroniside effectively downregulated the expression of the pro-inflammatory cytokines *IL-6* (Figure 2D), *TNF-α* (Figure 2E), and *IL-1β* (Figure 2F) while upregulating the expression of the anti-inflammatory cytokine *IL-10* (Figure 2G). Morroniside (50 μM) reversed the LPS-induced inhibition of superoxide dismutase (SOD) activity (Figure 2H), and, at all tested concentrations, markedly reduced the production of malondialdehyde (MDA) (Figure 2I). Immunofluorescence staining and flow cytometry analyses confirmed that morroniside (50 μM) reduced the levels of the pro-inflammatory activation marker CD86 (Figure 2J, K, and N), and increased the levels of the anti-inflammatory marker CD206 (Figure 2L-N).

**Figure 2:**
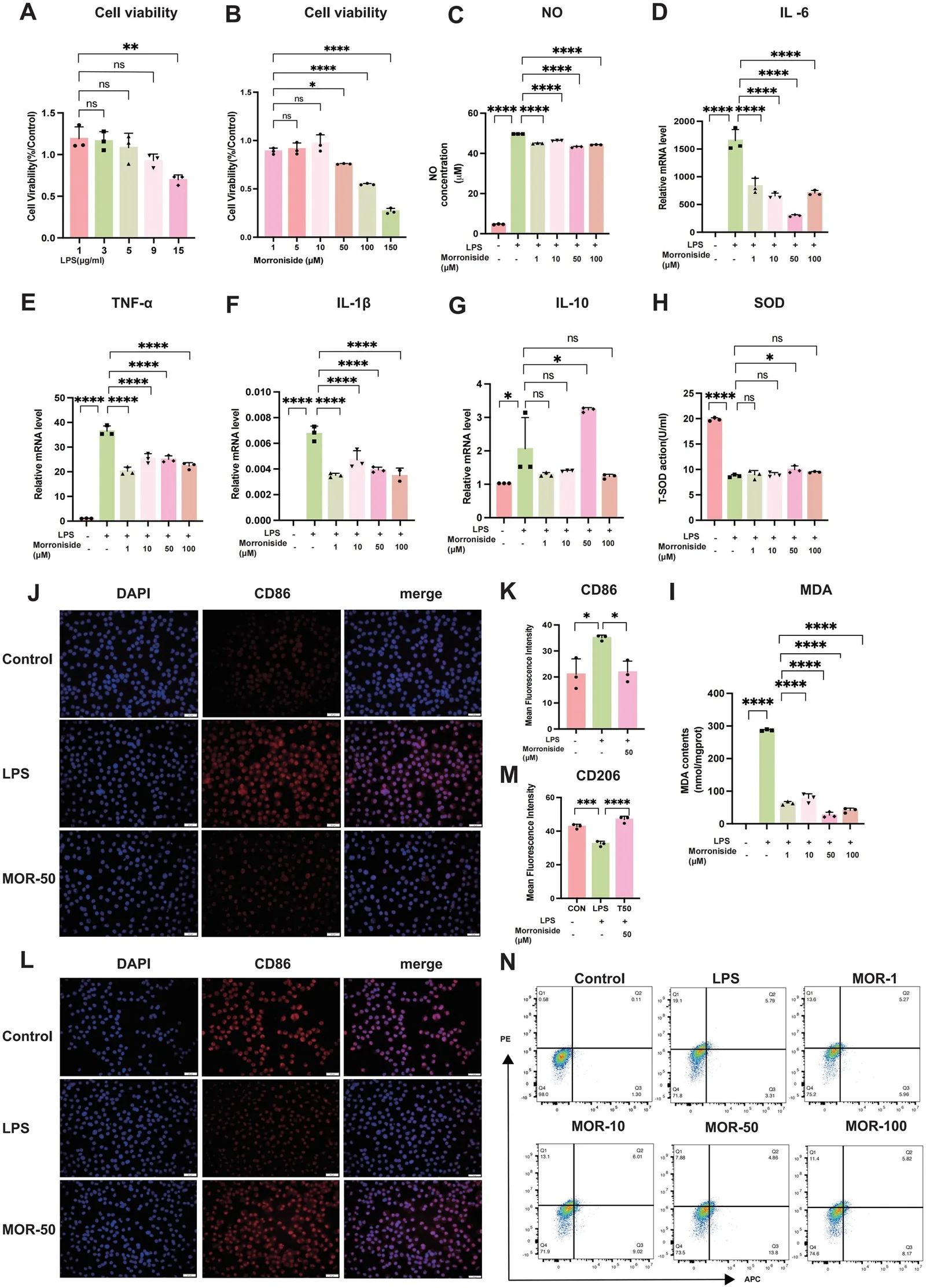
Morroniside attenuated the LPS-induced pro-inflammatory activation of BV2 cells and oxidative stress. (A, B) Cell viability of BV2 cells was assessed by CCK-8 assay after treatment with indicated concentrations of LPS (A) or morroniside (MOR) (B) for 24 hours. (C) NO levels in the culture supernatant were measured by Griess assay. (D-G) qRT-PCR method was used to quantify the transcriptional levels of pro-inflammatory cytokines (*IL-6, TNF-α, IL-1β*) and anti-inflammatory cytokines (*IL-10*). (H, I) Cellular SOD activity (H) and MDA content (I) were evaluated using commercial kits. (J-M) Representative immunofluorescence images (J, L) and quantitative analysis (K, M) of the pro-inflammatory marker CD86 and anti-inflammatory marker CD206. (N) Flow cytometric was used to detect the expression of PE stained-CD86 and APC stained-CD206. *P < .05, **P < .01, ***P < .001, ****P < .0001.

### Morroniside decreased ROS accumulation and maintained mitochondrial homeostasis after LPS treatment

To evaluate the extent of oxidative stress in pro-inflammatory microglia, we assessed total cell and mitochondrial ROS levels using flow cytometry and immunofluorescence. To assess mitochondrial damage, the mitochondrial membrane potential (ΔΨm) was evaluated by JC-1 staining, and the mitochondrial DNA copy number was evaluated based on *Nad2* expression.^23^ qRT-PCR results revealed that morroniside (1, 10, 50, and 100 μM) inhibited the expression of *Nd2* (Figure 3A). Flow cytometry revealed that morroniside (50 μM) decreased ROS production in BV2 cells induced by LPS (Figure 3B). Immunofluorescence staining of mitochondrial ROS showed that LPS stimulation promoted mitochondrial ROS production (Figure 3C and D), while morroniside (50 μM) treatment reversed this effect, consistent with the change in the total ROS levels induced by LPS (Figure 3B). Nrf2 translocases to the nucleus to exert its antioxidation effects. Immunofluorescence staining of Nrf2 revealed that LPS decreased Nrf2 nuclear translocation; however, morroniside (50 μM) significantly increased the nuclear Nrf2 level (Figure 3E and F). JC-1 flow cytometry results^24^ revealed that the mitochondrial membrane potential decreased significantly after LPS treatment of BV2 cells but morroniside (50 μM) reversed this decrease (Figure 3G). TEM was performed to directly visualize the mitochondrial ultrastructure and detect mitophagosome formation at the subcellular level.^25^ The TEM images showed increased mitochondrial swelling after LPS stimulation; morroniside (50 μM) treatment reduced the swelling and promoted mitophagy (Figure 3H).

**Figure 3:**
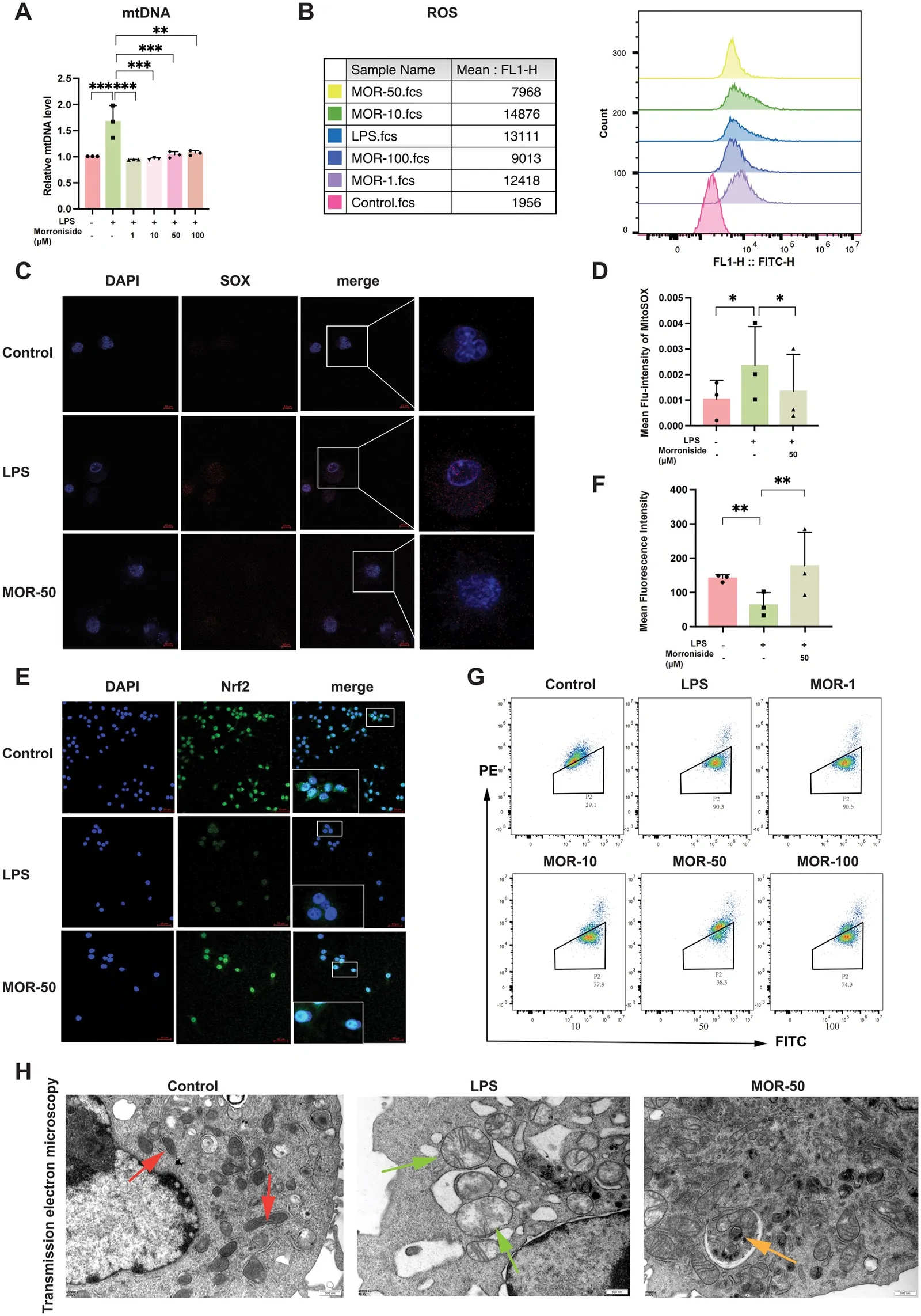
The impact of morroniside on the ROS accumulation and mitochondrial function after LPS treatment in BV2 cells. (A) mtDNA copy number was quantified using qRT-PCR. (B) Total intracellular ROS levels measured by flow cytometry using DCFH-DA probe. (C, D) Immunofluorescence staining was utilized to assess the mitochondrial ROS levels. (E, F) Immunofluorescence staining was deployed to detect the expression of Nrf2 in nuclei. (G) Mitochondrial membrane potential was determined by flow cytometry using JC-1 staining. A decrease in the red/green fluorescence ratio indicated depolarization. (H). Representative TEM images of mitochondria. Red arrows indicate normal mitochondrial; green arrows indicate swollen mitochondria; orange arrow indicates mitophagy. *P < .05, **P < .01, ***P < .001, ****P < .0001.

### Morroniside inhibits inflammation by upregulating the Nrf2/HO-1/p62 pathway and mitophagy-associated molecules and activating the NF-κB pathway

To define the mechanism underlying the anti-inflammatory, antioxidative, and pro-mitophagy effects of morroniside *in vitro*, we analyzed the levels of key proteins in the Nrf2/HO-1/NF-κB pathway and signature markers of mitophagy by WB. As shown in Figure 4A and B, LPS stimulation significantly decreased LC3 and TOM20 colocalization, which reflected mitophagosome formation, while morroniside (50 μM) treatment reversed this change. WB analyses revealed that morroniside enhanced the levels of nuclear Nrf2, HO-1, p62, LC3, Parkin, PINK1, and p-IκBa (Figure 4C-N, Q and R) while suppressing p-p65 levels (Figure 4O and P). These findings suggested that morroniside treatment upregulated the Nrf2/HO-1/p62 pathway and mitophagosome formation-associated molecules but suppressed NF-κB pathway activation.

**Figure 4:**
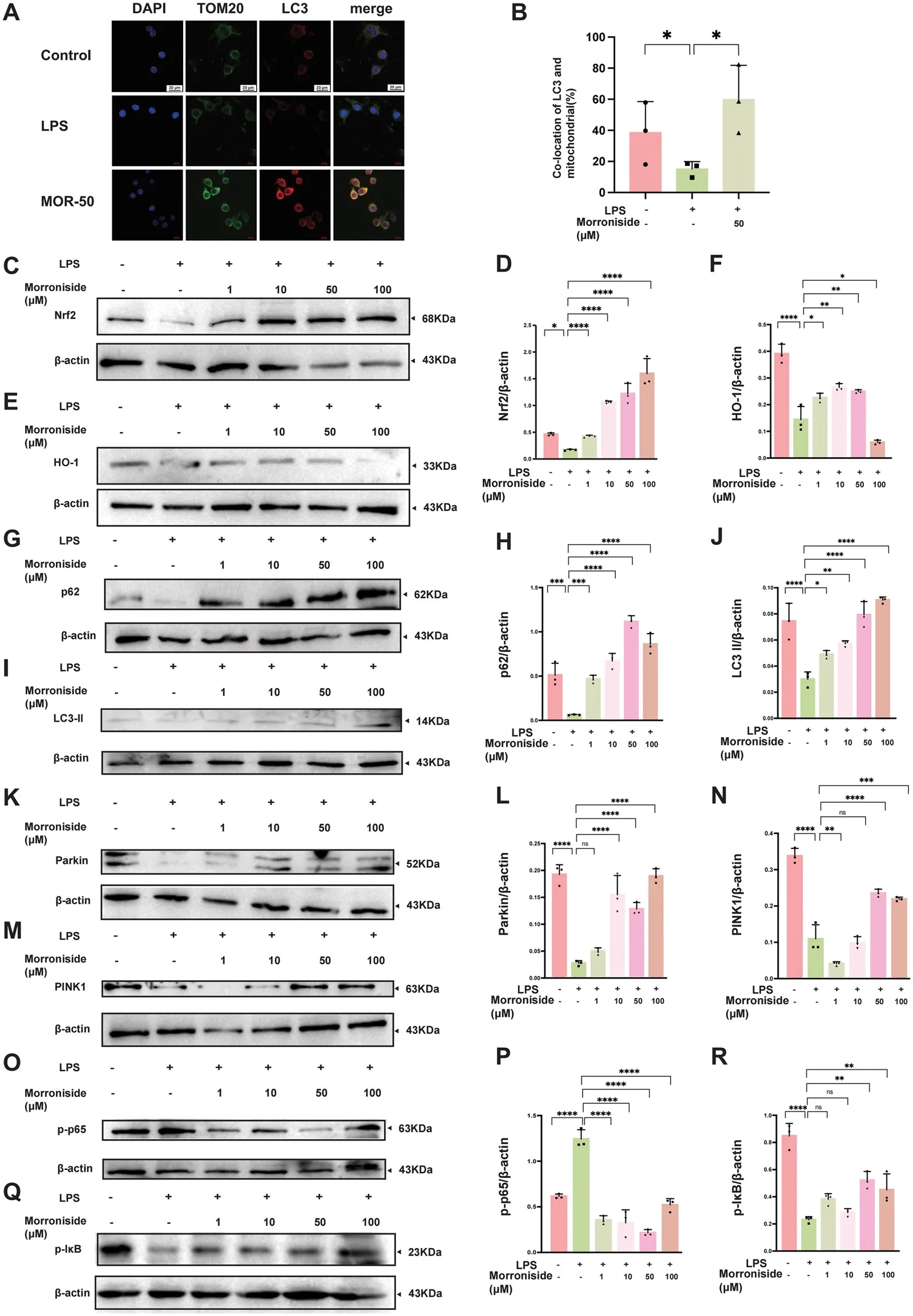
Morroniside activates the Nrf2/HO-1 axis, promotes mitophagy, and suppresses the NF-κB pathway in LPS-stimulated BV2 cells. (A, B) Representative immunofluorescence images (A) and quantitative analysis (B) show colocalization of LC3 and TOM20 indicating mitophagy. (C-R) Western blot analysis (C, E, G, I, K, M, O, Q) and corresponding quantitative densitometry (D, F, H, J, L, N, P, R) of key proteins involved in the Nrf2 antioxidant pathway (Nrf2, HO-1), mitophagy (p62, LC3-II, PINK1, Parkin), and the NF-κB inflammation pathway (p-p65, p-IκB). β-actin was used as a loading control. *P < .05, **P < .01, ***P < .001, ****P < .0001.

### Downregulation of Nrf2 inhibits the anti-inflammatory and anti-oxidative activity of morroniside *in vitro*

To investigate the function of Nrf2 in morroniside anti-inflammatory and antioxidative activity in BV2 cells, we downregulated *Nrf2* expression by transfecting BV2 cells with *Nrf2* siRNA before treatment with morroniside (50 μM) and LPS.^26–29^ WB and qRT-PCR analyses were used to determine the transfection efficiency, which showed that siRNA2- and siRNA3-transfected BV2 cells exhibited significant downregulation of *Nrf2* (Figure 5A-C). The siRNA2 sequence was selected to transfect BV2 cells in subsequent experiments. NO assay results indicated that morroniside (50 μM) inhibited NO production (Figure 5D) induced by LPS, but this effect was inhibited by *Nrf2* siRNA. qRT-PCR results showed that morroniside (50 μM) inhibited the expression of *IL-6* (Figure 5E), *TNF-α* (Figure 5F), and *IL-β* (Figure 5G) induced by LPS in BV2 cells, while *Nrf2* siRNA upregulated the expression of *IL-6*, *TNF-α*, and *IL-β*. *Nrf2* siRNA also reversed the increased expression of *IL-10* mediated by morroniside (Figure 2H). An MDA assay revealed that morroniside (50 μM) suppressed MDA production, while *Nrf2* siRNA blocked this effect (Figure 5I). A SOD assay indicated that *Nrf2* siRNA reversed the SOD-promoting effect of morroniside induced by LPS (Figure 5J). Flow cytometry showed that morroniside (50 μM) decreased ROS production and increased the mitochondrial membrane potential; *Nrf2* siRNA reversed these effects (Figure 5K and L). Immunofluorescence staining and flow cytometric analyses demonstrated that CD86 downregulation and CD206 upregulation induced by morroniside were also reversed by *Nrf2* siRNA (Figure 5M-Q).

**Figure 5:**
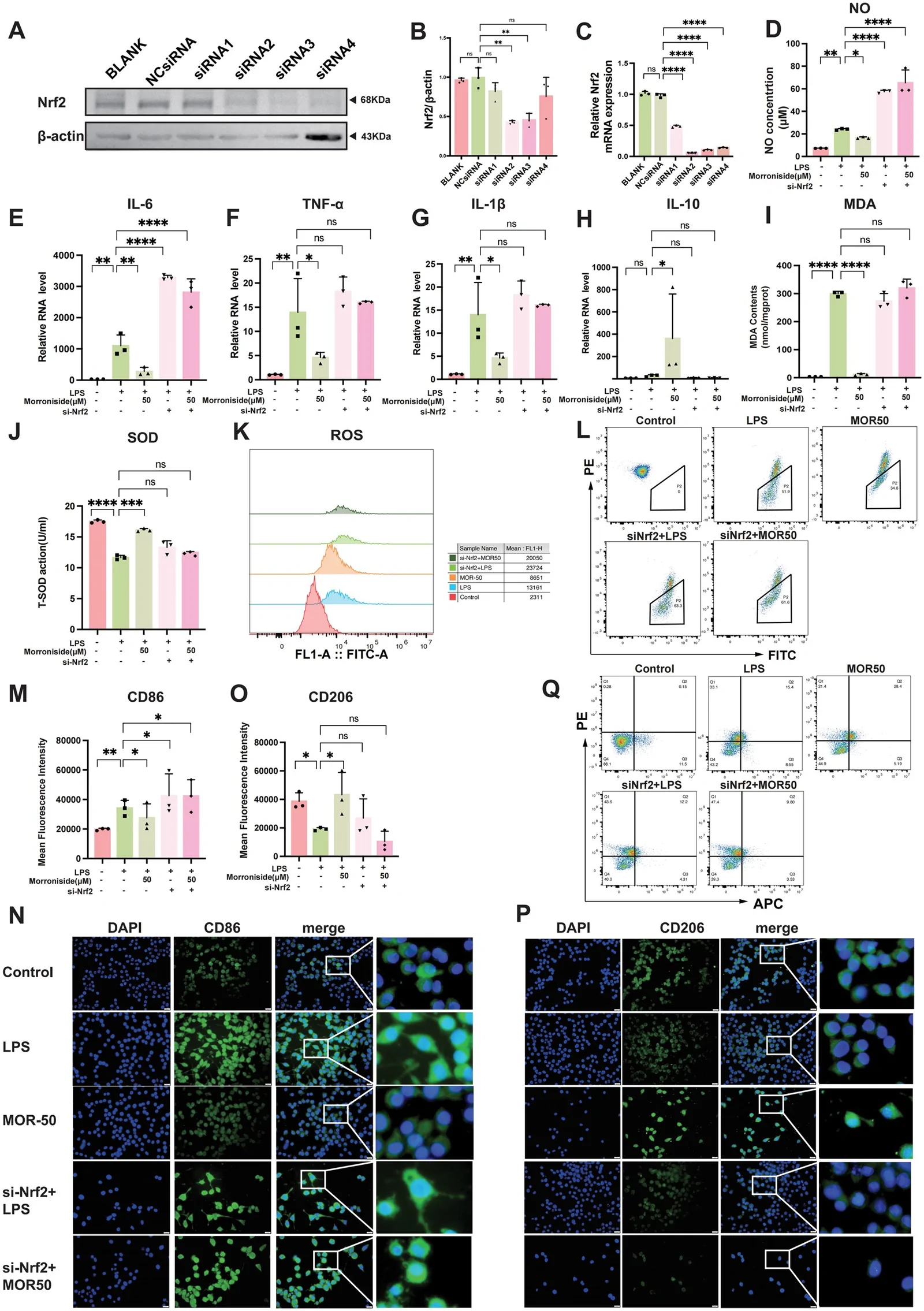
Nrf2 siRNA reversed the anti-inflammatory and antioxidant effects of morroniside in LPS-stimulated BV2 cells. (A-C) siRNA transfected efficiency was assessed using Western Blot (A, B) and qRT-PCR (C). (D) NO levels in the culture supernatant were measured by Griess assay. (E-H) qRT-PCR method was used to quantify the transcriptional levels of pro-inflammatory cytokines (*IL-6*, *TNF-α*, *IL-1β*) and anti-inflammatory cytokines (*IL-10*). (I, J) Cellular MDA content (I) and SOD activity (J) were evaluated. (K) Total intracellular ROS levels were measured by flow cytometry using DCFH-DA. (L) Mitochondrial membrane potential was determined by flow cytometry using JC-1 staining. (M-Q) Immunofluorescence staining and flow cytometry were utilized to assess the expression of CD86 and CD206. *P < .05, **P < .01, ***P < .001, ****P < .0001.

### Downregulation of Nrf2 inhibits morroniside promotion of mitophagosomes and NF-κB suppression *in vitro*

The crucial functions of Nrf2 were further demonstrated by a decrease in morroniside-induced LC3/TOM20 colocalization following *Nrf2* siRNA administration in BV2 cells (Figure 6A and B). WB analysis showed that *Nrf2* downregulation decreased the levels of HO-1, p62, LC3, Parkin, and p-IκB (Figure 6C-L), which were regulated by morroniside treatment. Collectively, these findings demonstrate that Nrf2 orchestrated the multifaceted therapeutic actions of morroniside, including anti-inflammation, antioxidation, mitophagy enhancement, and NF-κB pathway suppression.

**Figure 6:**
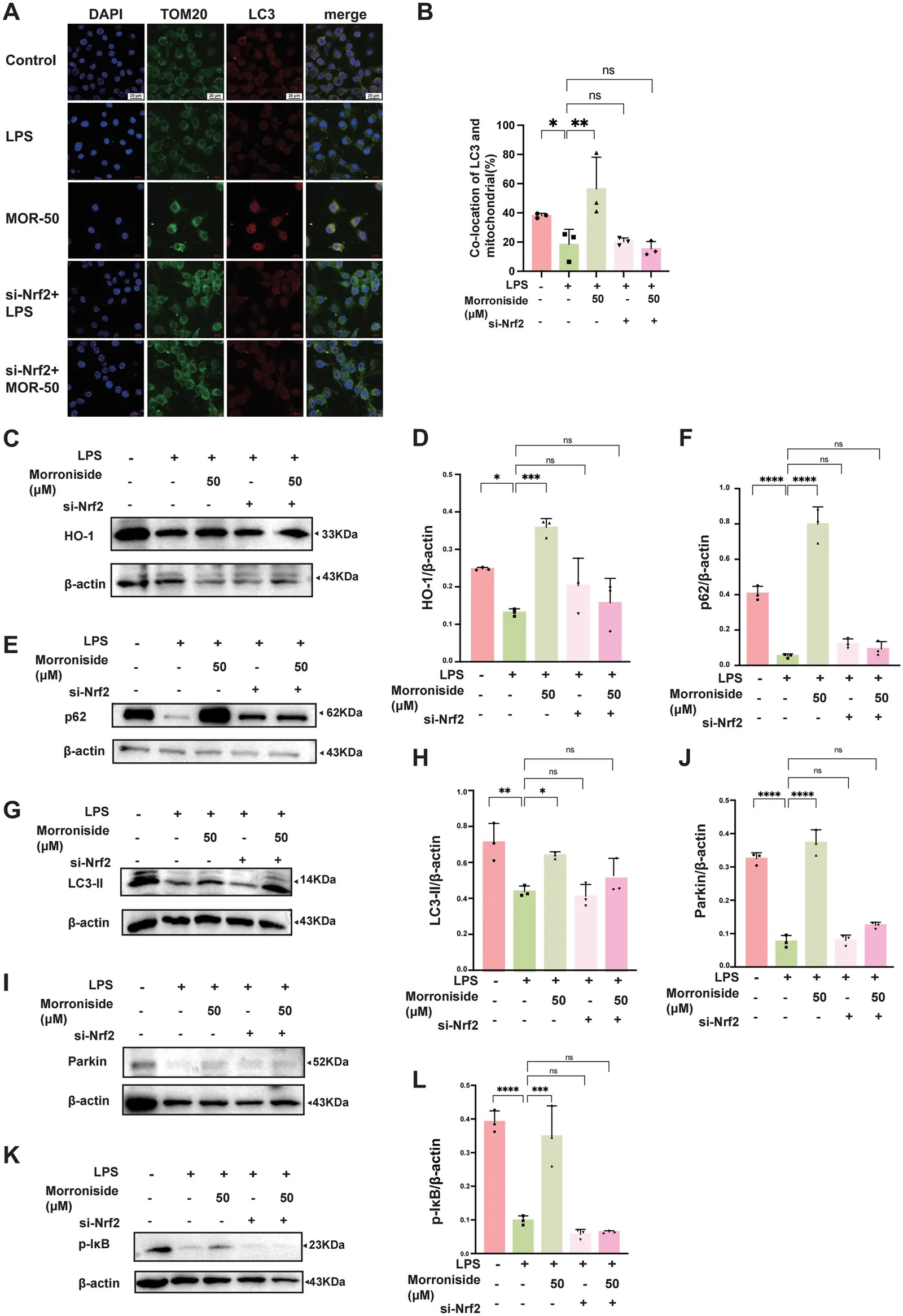
Nrf2 siRNA reversed morroniside-induced promotion of mitophagy and inhibition of NF-κB activation in LPS-stimulated BV2 cells. (A, B) Representative immunofluorescence images (A) and quantitative analysis (B) showing the colocalization of LC3 and TOM20 indicating mitophagy. (C-L) Western blot analysis (C, E, G, I, K) and corresponding quantitative densitometry (D, F, H, J, L) of key proteins in the mitophagy pathway (p62, LC3-II, PINK1) and the NF-κB pathway (p-p65, p-IκB). β-actin was used as a loading control. *P < .05, **P < .01, ***P < .001, ****P < .0001.

### Morroniside attenuates EAE in C57BL/6 mice

To evaluate the therapeutic efficacy of morroniside *in vivo*, we established an EAE mouse model, a well-established model of MS. Morroniside was administered on day 7 post-immunization and disease progression was monitored by daily clinical scoring and body weight measurements (Figure 7A). As shown in Figure 7B, mice with EAE treated with morroniside (100 mg/kg) before the onset of clinical disease had significantly decreased clinical scores and lower body weight losses throughout the study period compared to those that did not receive morroniside (Figure 7B and C). Behavioral assessments, including the open field and beam walking tests, were conducted on day 21 post-immunization. The open field test results revealed that morroniside (100 mg/kg) treatment attenuated the decrease in total distance caused by EAE (Figure 7D and E). The beam walking test^30–32^ showed that mice with EAE had an increased crossing time and number of foot slips compared to control mice, while morroniside treatment decreased these parameters (Figure 7F and G). Furthermore, histological analysis, including H. and E. and MBP staining, was conducted to investigate inflammatory cell infiltration and demyelination in spinal cord tissue. Mice with EAE developed inflammatory cell infiltration in the CNS, while morroniside (100 mg/kg) treatment lessened the infiltration (Figure 7H and I). Moreover, immunofluorescence staining of the brain and lumbar spinal cord tissue demonstrated that there was less MBP immunofluorescence intensity, reflecting demyelination, in mice with EAE but morroniside treatment (100 mg/kg) increased the MBP staining intensity (Figure 7J-L).

**Figure 7:**
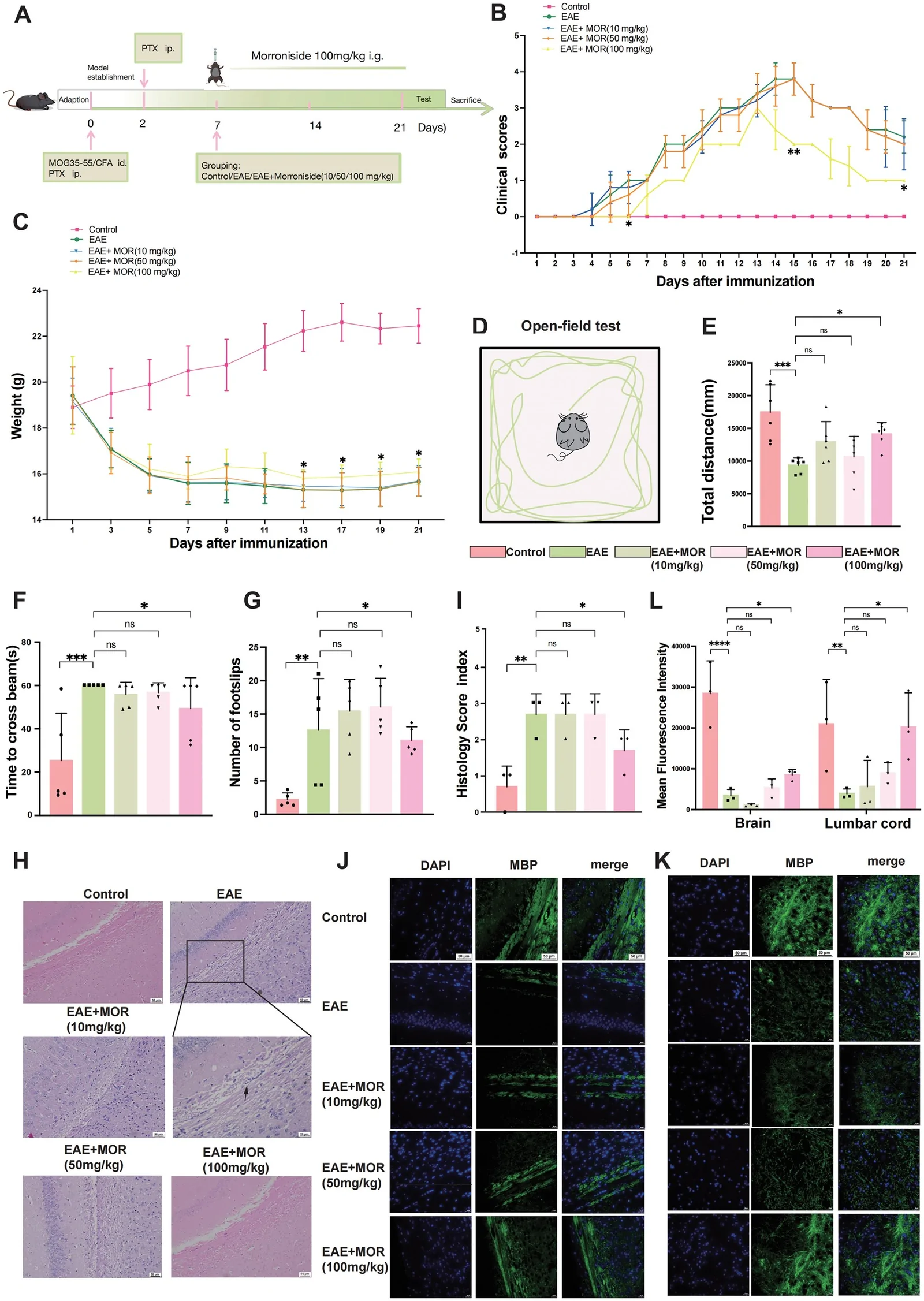
Morroniside treatment improved neurological function and attenuated inflammation and improved demyelination in mice with EAE. (A) Scheme of the EAE model establishment and morroniside treatment. (B, C) Clinical scores (B) and body weight changes (C) of mice were recorded daily after immunization. (D, E) The open field test assessed the total distance covered. (F, G) Beam walking test assessed the total cross time and foot slips. (H, I) Representative images (H) and quantitative analysis (I) of inflammatory cell infiltration in the corpus callosum by H&E staining. Arrow pointed to the perivascular cuffs. (J-L) Representative immunofluorescence images and quantitative analysis of myelin basic protein (MBP) expression (L) in the corpus callosum (J) and lumbar spinal cord (K). *P < .05, **P < .01, ***P < .001, ****P < .0001.

### Morroniside alleviates microglial activation and oxidative stress in mice with EAE

To elucidate the molecular mechanism underlying the morroniside-mediated improvement of neurological deficits in EAE, we assessed oxidative stress and inflammatory markers in brain and spinal cord tissues of the mice. A SOD assay indicated that morroniside (100 mg/kg) partially restored SOD production that had been reduced by EAE in both the brain and lumbar spinal cord (Figure 8A). An MDA assay revealed that morroniside suppressed MDA production in the brain and lumbar spinal cord (Figure 8B). qRT-PCR results showed that morroniside treatment inhibited the EAE-induced transcription of *IL-6* (Figure 8C), *TNF-α* (Figure 8D), and *IL-β* (Figure 8E). Furthermore, *IL-10* transcription was increased by morroniside (Figure 8F). ELISA results were consistent with the qRT-PCR results (Figure 8G–I). Immunofluorescence staining demonstrated that morroniside downregulated CD86 and upregulated CD206 in EAE brain and lumbar spinal cord tissue (Figure 8J–O). Collectively, these results illustrated that morroniside induced anti-inflammatory and antioxidative effects on microglia *in vivo*.

**Figure 8:**
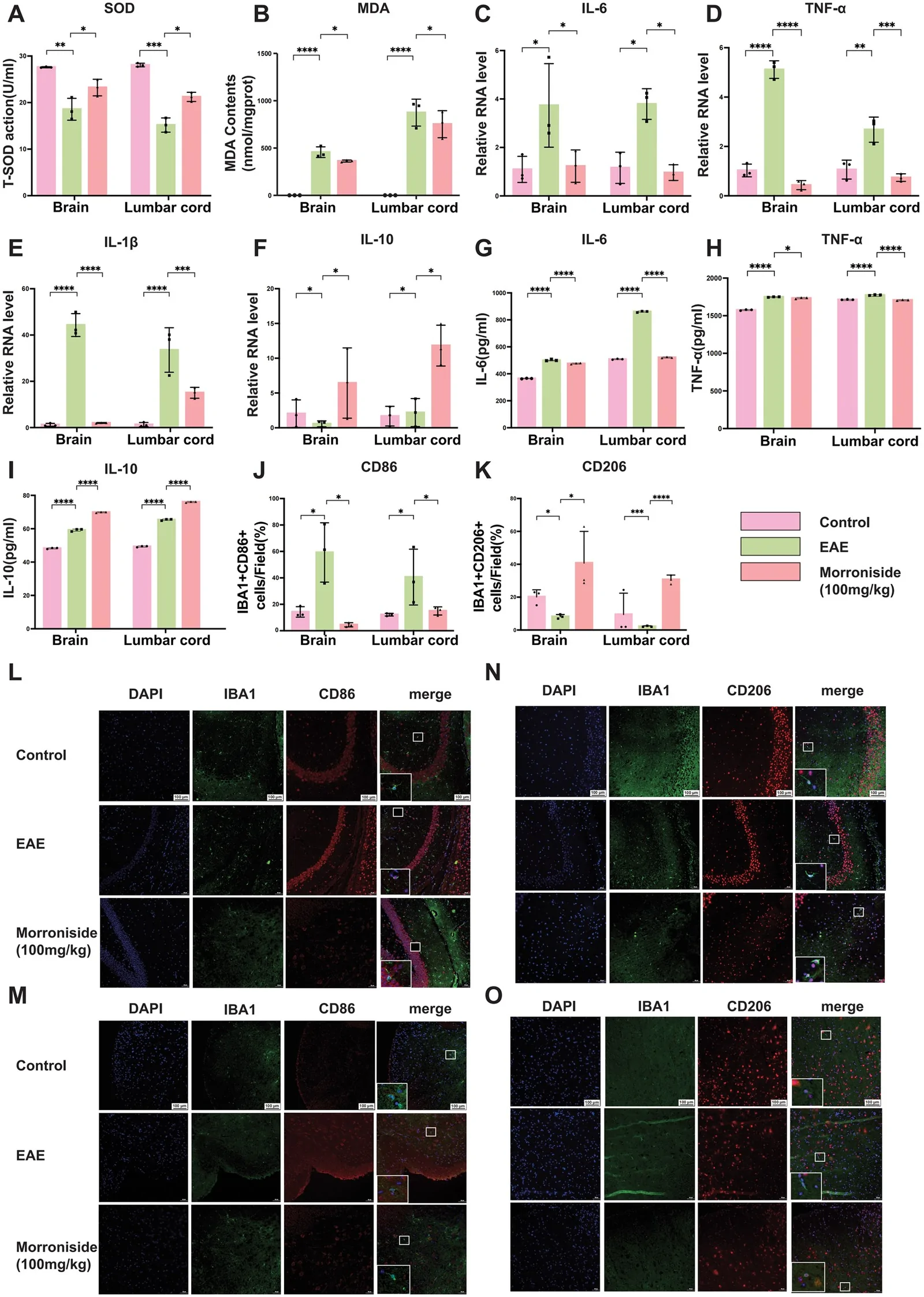
Morroniside alleviated microglial activation and oxidative stress in EAE mice. (A, B) The activity of SOD (A) and the content of MDA (B) in brain and lumbar spinal cord were measured. (C-F) qRT-PCR method was used to quantify the transcriptional levels of pro-inflammatory cytokines (*IL-6*, *TNF-α*, *IL-1β*) and anti-inflammatory cytokines (*IL-10*) in brain and lumbar cord. (G-I) An ELISA kit was employed to quantify the IL-6, TNF-α, and IL-10 concentrations in tissue supernatant. (J-O) Representative immunofluorescence images (L-O) and quantitative analysis (J, K) of the pro-inflammatory marker CD86 and the anti-inflammatory marker CD206 in brain and the spinal cord. *P < .05, **P < .01, ***P < .001, ****P < .0001.

### Modulation by morroniside of the Nrf2/HO-1/p62 pathway and mitophagy in EAE mice

Finally, we explored the involvement of the Nrf2/HO-1/p62 pathway, mitophagy, and NF-κB pathway in EAE. As shown in Figure 9A and C, LC3 and TOM20 colocalization in microglia was decreased in mice with EAE but this effect was significantly reversed by morroniside treatment. Morroniside effectively reduced the number of TFAM-positive microglia induced in the hippocampal regions (Figure 9B and D). The increase in TFAM-positive microglia indicated a greater amount of mtDNA released in the brain tissue.^33^ The qRT-PCR results showed that the transcriptional expression of *Nrf2* increased in the brain and spinal cord tissues in EAE but *HO-1* expression was decreased (Figure 9E and F). Brain and spinal cord of mice with EAE showed decreased levels of Nrf2; this was reversed by morroniside treatment (Figure 9G). The protein levels of HO-1 were consistent with the qRT-PCR results (Figure 9H). Consistent with our *in vitro* findings, p62, LC3, Parkin, PINK1, and p-IκB levels (Figure 9I-L and N) were depressed in the brain and spinal cord of mice with EAE while p-p65 (Figure 9M) levels increased; these changes were significantly reversed by morroniside treatment. Taken together, these results indicate that morroniside markedly upregulated the Nrf2/HO-1/p62 pathway, which enhanced mitophagy and suppressed NF-κB pathway phosphorylation, thereby ameliorating neuroinflammation and oxidation of microglia in EAE.

**Figure 9:**
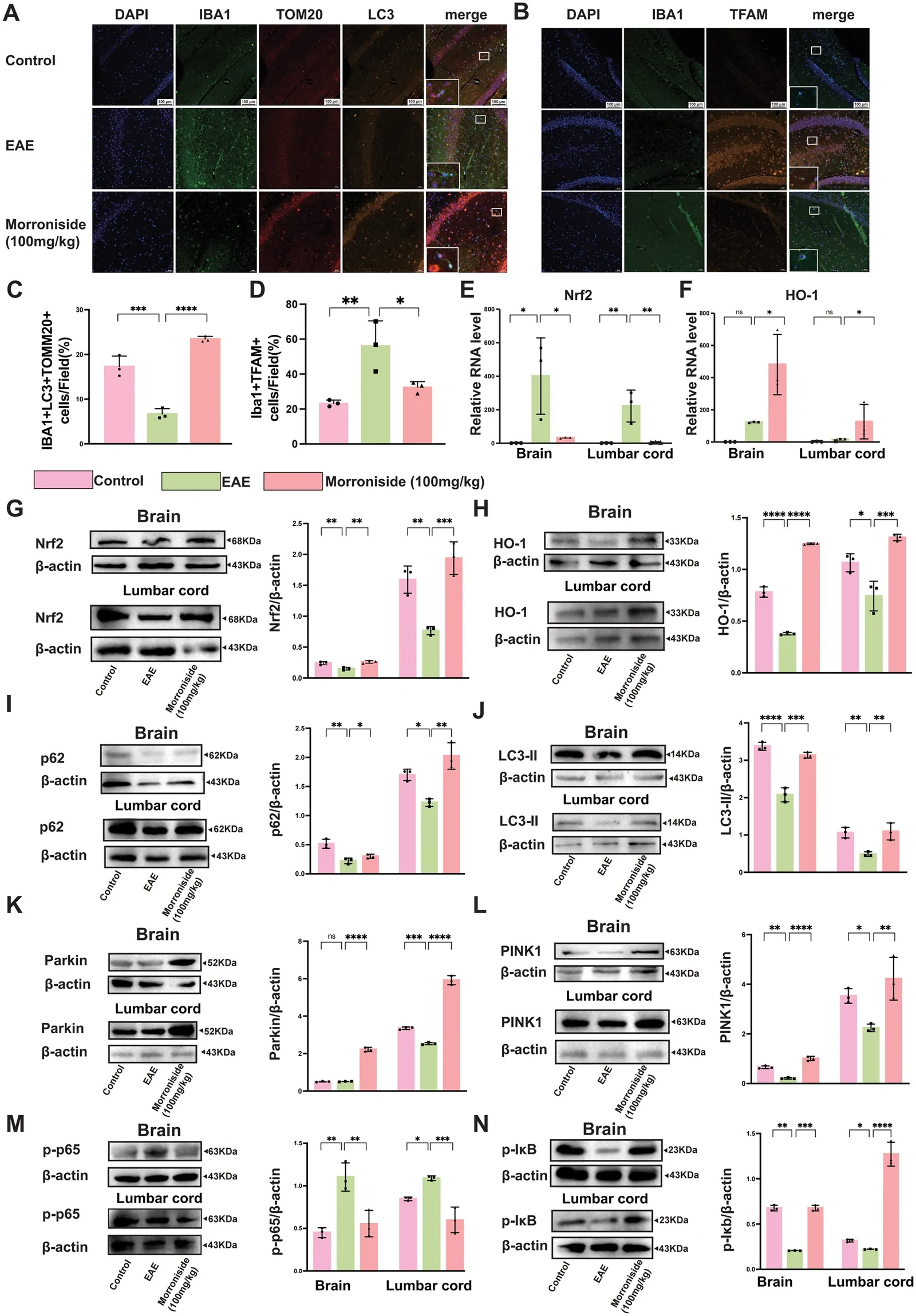
Morroniside activated Nrf2/HO-1/p62 axis and enhanced mitophagy in mice with EAE. (A-D) Representative immunofluorescence images and quantitative analysis showing the colocalization of LC3 with TOM20 (A, C) and TFAM (B, D) with the microglial marker Iba1 in brain, indicative of microglial mitophagy and mitochondrial biogenesis level. (E, F) qRT-PCR method was used to quantify the transcriptional levels of *Nrf2* and *HO-1* in brain and lumbar cord. (G-N) Western blot analysis and corresponding quantitative densitometry of key proteins in the Nrf2 pathway (Nrf2, HO-1) (G, H), mitophagy pathway (p62, LC3-II, Parkin, PINK1) (I-L), and NF-κB pathway (p-p65, p-IκB) (M, N) in brain and spinal cord tissues. β-actin served as a loading control. *P < .05, **P < .01, ***P < .001, ****P < .0001.

## Discussion

Microglia, the resident immune cells of the CNS, are fundamental for maintaining CNS inflammatory homeostasis. In EAE, however, aberrantly and persistently activated microglia are known drivers of pathological neuroinflammation and disease progression,^13^^,^^34^ and are, therefore, important potential therapeutic targets. Recently, there has been growing interest in developing therapeutic interventions in MS patients using traditional Chinese medicine, to modulate microglial activity toward an anti-inflammatory phenotype.^35^ Morroniside is a promising candidate for MS treatment. Previous studies have shown that it has favorable oral bioavailability, can cross the blood-brain barrier, and has potent anti-inflammatory and antioxidative efficacy in the CNS of mice with EAE.^36–38^ Our group previous studies have also confirmed that morroniside treatment ameliorated the clinical signs of EAE and mitigated neuroinflammation through modulating the balance of peripheral Th1, Th17, and regulatory T (Treg) cells.^38^ However, as a natural product, morroniside may exert multiple biological activities. This study specifically focused on elucidating its anti-inflammatory effects in microglia. In this research, the coordinated suppression of pro-inflammatory markers (IL-1β, TNF-α, IL-6, NO, and CD86) and concomitant upregulation of anti-inflammatory markers (IL-10, CD206) in both the LPS-induced BV2 cell model and the EAE mouse model confirmed that morroniside could promote a shift in microglia from a pro-inflammatory state to an anti-inflammatory state. To further investigate the nderlying mechanism of morroniside in modulating the microglial inflammatory state, we conducted experiments based on the prominent antioxidant properties of morroniside.

The antioxidative capacity of morroniside was evidenced by a marked reduction in ROS, mtROS, and lipid peroxidation (MDA), coupled with the restoration of the mitochondrial membrane potential and the coordinated Nrf2 activation in microglia. This aligned with the master antioxidant regulator role of Nrf2, which, once activated, dimerizes with sMAF protein to bind to AREs in the nucleus and drive the transcription of antioxidant genes, including *HO-1* and *SOD.*^39^^,^^40^ Furthermore, the anti-inflammatory and anti-oxidant effect was entirely reversed by *Nrf2* siRNA, unequivocally identifying Nrf2 as the central molecular target in the action of morroniside. These findings validated our initial network pharmacology predictions. Nrf2 indeed has an essential function in multiple diseases, including diabetes, cardiac injury, cancers, and neurodegenerative disease.^41^^,^^42^ The Nrf2 activator dimethyl fumarate is already FDA-approved for MS therapy due to its antioxidant effects through Nrf2 signaling.^43^ However, with continued research, more studies have provided evidence that Nrf2 participates in both antioxidant signaling pathways and mitophagy, an autophagic mechanism by which dysfunctional mitochondria are degraded.^42^ Furthermore, emerging evidence has shown that mitophagy and inflammation may interact in several pathological events and that mitophagy dysfunction may exacerbate inflammation.^44^ Specifically, mitochondrial dysfunction induced by various insults, including LPS, morphine, and cocaine stimuli, is a key source of ROS and mtDNA, which can trigger multiple inflammatory cascades via NF-κB, NLRP3, and the CGAS-STING1 pathways. The removal of damaged mitochondria by mitophagy is therefore essential for resolving neuroinflammation.^13^^,^^45^ The most comprehensively investigated mitophagy mechanism is the ubiquitin-dependent mitophagy pathway,^46^^,^^47^ which involves the translocation of PTEN-induced putative kinase 1 (PINK1) from the outer mitochondrial membrane to the mitochondrial matrix, the phosphorylation and recruitment of cytosolic parkin RBR E3 ubiquitin protein ligase (PRKN/PARK2), and the recruitment of autophagic receptor proteins SQSTM1/p62 and MAP1LC3B/LC3B.^48–50^ Our *in vitro* and *in vivo* WB analyses demonstrated that morroniside activated the Nrf2/HO-1 antioxidant pathway, enhanced p62/Parkin/PINK1-dependent mitophagy, as evidenced by increased levels of PINK1, Parkin, and LC3-II, enhanced colocalization of LC3 with the mitochondrial protein TOM20, and restored ultrastructural integrity in electron microscopy images and suppressed NFκB-mediated inflammation. The mechanism is schematically depicted in Figure 10.

**Figure 10:**
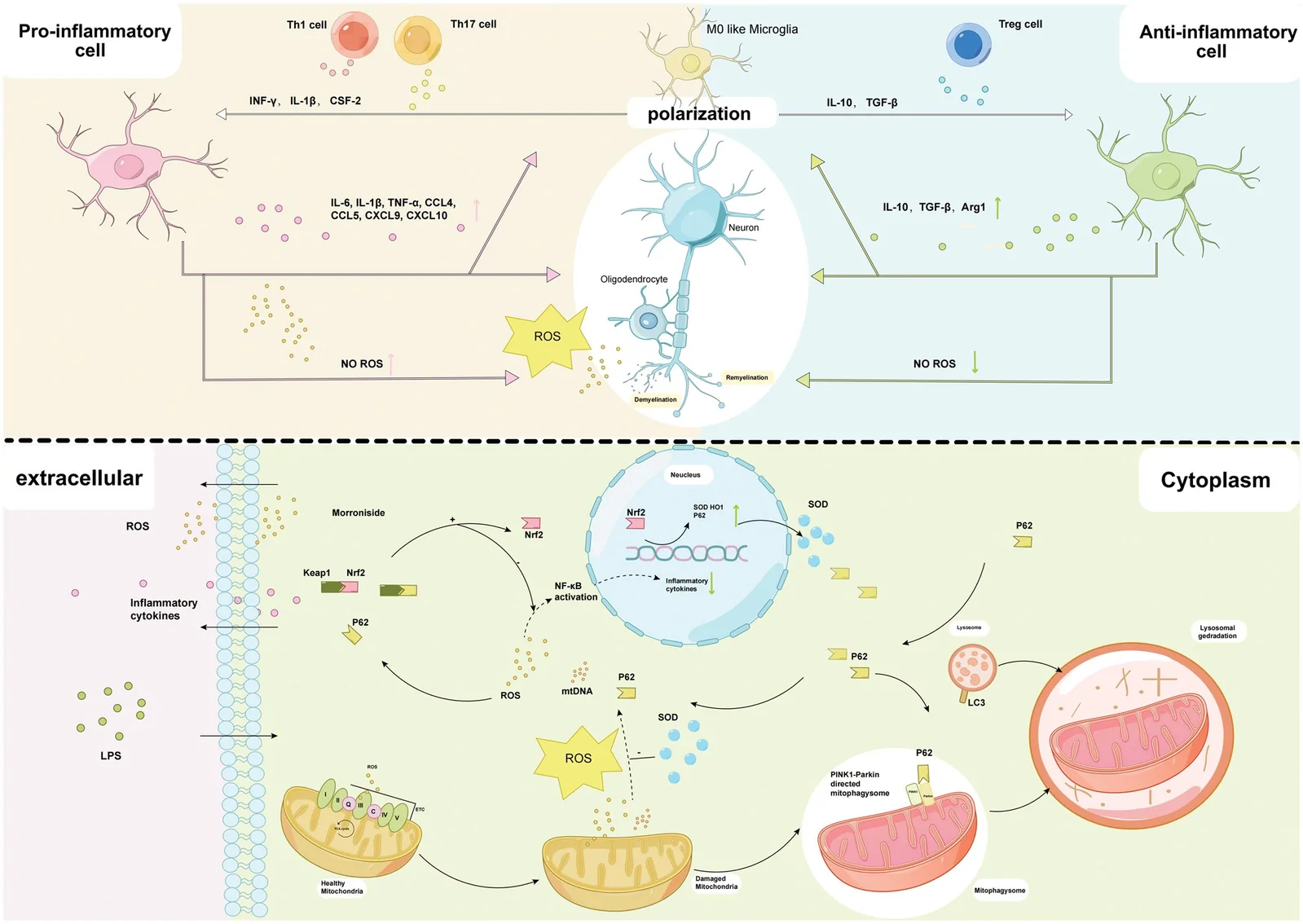
Mechanistic schema of morroniside-mediated microglial polarization via Nrf2/HO-1/p62 axis in EAE. This schematic illustrates the regulatory effects of morroniside on microglial phenotype shifting and mitochondrial homeostasis. (1) Pro-inflammatory microglia were activated by Th1/Th17 cell to secret inflammatory cytokines (IL-6, IL-1β, TNF-α, CCL4, CCL5, CXCL9, CXCL10) and ROS to amplify the inflammation and attack myelin. (2) Anti-inflammatory microglia were activated by Treg cell characterized by IL-10/TGF-β/Arg-1 secretion, ultimately protecting oligodendrocytes from demyelination. (3) Mitochondrial oxidative stress was aggravated by elevated ROS and impaired mitochondria. Excess ROS and mtDNA released from dysfunctional mitochondria triggered NF-κB signaling activation leading to inflammation. (4) Morroniside treatment could activate Nrf2 nuclear translocation. This upregulated HO-1 to scavenge ROS and enhance SOD activity, simultaneously upregulating p62 to enhance PINK1/Parkin-dependent mitophagy. The clearance of damaging ROS and mtDNA alleviates inflammation, facilitating the shift of microglia toward an anti-inflammatory, protective phenotype.

Notably, whereas the established MS therapy dimethyl fumarate primarily engages Nrf2-dependent antioxidant responses,^43^ our study shows that morroniside activates Nrf2 to coordinate regulation of the “oxidation-mitochondrial dysfunction-inflammation” cycle in microglia. A key mechanistic insight revealed in this study was the positive feedback loop between Nrf2 and the autophagy receptor p62. Upon activation, Nrf2 transactivated the expression of *p62* by binding to AREs in its promoter region, thereby enhancing mitophagy.^41^ Additionally, p62 and Keap1 competitively bind to the ETGE motif of Nrf2, which disrupts the Keap1-mediated ubiquitination and degradation of Nrf2, leading to its stabilization and further transcriptional activity.^42^^,^^51^^,^^52^ Therefore, p62 is a critical molecular bridge that functionally connects the Nrf2-mediated antioxidant response to mitophagy execution. This self-amplifying Nrf2/p62 circuit is crucial for efficiently clearing damaged mitochondria, and it provides a unified means of mitigating oxidative stress, suppressing inflammation, and restoring mitochondrial homeostasis.^53–56^ This integrated mechanism represents a novel approach to restore microglial homeostasis in neuroinflammation.

Our study has several limitations. First, although the *Nrf2* siRNA *in vitro* experiments robustly supported the essential role of Nrf2 in driving mitophagy and microglial anti-inflammatory regulation, further validation using microglia-specific *Nrf2* knockout models or adenovirus-mediated gene manipulation *in vivo* would strengthen our findings. Second, although we identified NF-κB as a downstream pathway, mitophagy may also modulate inflammation through alternative pathways, such as STING or inflammasome signaling, which remain to be explored.^57^^,^^58^ Third, the reliance on murine BV2 microglia, while experimentally convenient, may not fully recapitulate human microglial biology, and experiments in future studies should use human-derived microglia. Fourth, although we focused on Nrf2 as a central target, the polypharmacological targets of herbal compounds suggest that morroniside may engage additional molecular networks. Fifth, the effects on peripheral mononuclear cells were not investigated on peripheral mononuclear cells in this work. Future studies should use microglia-specific conditional knockout models to dissect the central versus peripheral mechanisms of the actions of morroniside. Finally, our findings demonstrated the therapeutic effects of morroniside in the EAE model. However, this model primarily replicates acute CNS inflammation and does not fully capture key aspects of human MS pathology, such as chronic disease progression and remyelination failure. Therefore, our findings should be interpreted within this specific preclinical context. It is also important to note that these preclinical results require further validation and the translation to clinical applications in MS patients remains a future endeavor.

## Conclusion

In summary, this study demonstrated that morroniside alleviated EAE by coordinating an integrated protective mechanism linking Nrf2/HO-1-driven antioxidant signaling to p62/PINK1/Parkin-mediated mitophagy, ultimately reprogramming microglia toward an anti-inflammatory phenotype. These findings provide mechanistic insights into the neuroprotective properties of morroniside and underscore the restoration of mitochondrial homeostasis as a promising therapeutic intervention for autoimmune demyelinating disorders such as MS.

## Data Availability

All data supporting this study are included in the article and its supplementary materials.
